# Fuel Use and Greenhouse Gas Emissions from Offshore Fisheries of the Republic of Korea

**DOI:** 10.1371/journal.pone.0133778

**Published:** 2015-08-28

**Authors:** Jeong-A Park, Caleb Gardner, Myo-In Chang, Do-Hoon Kim, Young-Soo Jang

**Affiliations:** 1 Institute for Marine and Antarctic Studies, University of Tasmania, Hobart, Tasmania, Australia; 2 Department of Marine and Fisheries Business and Economics, Pukyong National University, Busan, Republic of Korea; 3 Ministry of Oceans and Fisheries Korea, Sejong, Republic of Korea; Centro de Investigacion Cientifica y Educacion Superior de Ensenada, MEXICO

## Abstract

Greenhouse Gas (GHG) emissions from the offshore fisheries industry in the Republic of Korea (Korea) were examined in response to growing concerns about global warming and the contribution of emissions from different industrial sectors. Fuel usage and GHG emissions (CO_2_, CH_4_, N_2_O) were analysed using the ‘Tier 1’ method provided by the Intergovernmental Panel on Climate Change (IPCC) from the offshore fishery, which is the primary domestic seafood production sector in Korea. In 2013, fuel usage in the offshore fishery accounted for 59.7% (557,463 KL) of total fuel consumption of fishing vessels in Korea. Fuel consumption and thus GHG emissions were not stable through time in this industry, increasing by 2.4% p.a. for three consecutive years, from 2011 to 2013, despite a decrease in the number of vessels operating. GHG emissions generated in offshore fisheries also changed through time and increased from 1,442,975 tCO_2_e/year in 2011 to 1,477,279 tCO_2_e/year in 2013. Changes in both fuel use and GHG emissions per kg offshore fish production appeared to be associated with decreasing catch rates by the fleet, which in turn were a reflection of decrease in fish biomass. Another important feature of GHG emissions in this industry was the high variation in GHG emission per kg fish product among different fishing methods. The long line fishery had approximately three times the emissions of the average production while the jigging fishery was more than two times higher than the average. Lowest emissions were from the trawl sector, which is regarded as having greatest environmental impact using traditional biodiversity metrics although had lowest environmental impact in terms of fuel and GHG emission metrics used in this study. The observed deterioration in fuel efficiency of the offshore fishery each year is of concern but also demonstrates that fuel efficiency can change, which shows there is opportunity to improve efficiency with changes to fishery management and harvesting operations.

## Introduction

The Korean government has expressed a desire to reduce Greenhouse Gas (GHG) emissions from industries including fisheries due to rising concern about Global Warming (Korean Ministry of Environment, 2014). Extensive analysis of GHG emissions from fisheries has been conducted in part as a response to GHG emission targets that are binding under international law by the Kyoto Protocol [1, 2, 3, 4].

The Republic of Korea (Korea) has committed to voluntary restrictions, which has led to strengthening of the country’s GHG governance. This has included annual reduction targets and an allocation plan for GHG enacted in September 2014 [5]. Korea has also established the “Korean Allowance Unit” (KAU) to control GHG from domestic industry with a target from 2015 to 2017 of 1,687 million KAU [5], which is equivalent to 1,687 million tCO_2_e (tons of carbon dioxide equivalent).

Although fisheries were not included as a mandated industry in the first three years of the GHG allocation plan, the reduction of GHG emissions remains a significant issue for Korean fisheries because the Korean government has set targets to reduce GHG emissions by 5.2% below business-as-usual (BAU) levels by 2020 [5]. Furthermore, it is possible that the level of GHG emissions from the fisheries industry will impact on seafood exports in response to increased global awareness in protecting the environment [6].

Precise measurement of GHG emissions is an important first step in management and several studies have been conducted to evaluate GHG emissions generated by fisheries in Korea [7, 8, 9, 10]. However, accurate measurement of the GHG is complicated by diversity in some fishing methods [8, 10], and also assessment methods. Examples of method issues with GHG emissions include the use of only carbon dioxide for the total GHG emissions, the application of stationary combustion factor instead of mobile combustion factor in the GHG calculations [7, 9], and using prior version of net calorific values of the fuel type based on the Energy Act standards which were amended in December 2011 in Korea [7, 8, 9].

The quantitative analysis presented here shows fuel usage and GHG emissions from Korea’s largest fishery sector, the offshore fishery, which covers operations with trip lengths of greater than one day [11]. Many fishing method categories are used including larger industrial methods such as purse seine and benthic trawl although more artisanal methods such as dive are also part of the offshore fishery. The aim of this analysis of the offshore fisheries of Korea was to quantify the scale of fuel use and emissions from different components of the fishery and also to determine if there were any processes resulting in change between those recent years where data were available. This was intended to provide insight on whether there were opportunities to address the government’s desire to reduce emissions.

Amongst the diverse range of fisheries in Korea, the offshore fishery is of special interest because it is the largest contributor to Korean domestic seafood production and likely to be most impacted by regulation due to a higher proportion of fuel and energy use [12]. Changes in this Korean industry is also of interest because fisheries have unusually high cultural value in Korea with per capita consumption (60.4 kg) more than three times the global average (18.9 kg) [13] and 1.7% of the global catch taken by this nation with only 0.7% of the global population [14].

We determined GHG emissions from this fishery using the ‘Tier 1’ method and also applied water-borne navigation emission factors which are included in mobile combustion emission factors. Both are provided by the Intergovernmental Panel on Climate Change (IPCC). This study estimated the emissions of carbon dioxide (CO_2_), methane (CH_4_) and nitrous oxide (N_2_O), which typically account for 95 percent of energy system emissions and are largely driven by the combustion of fossil fuels as GHG emissions [15].

### Domestic fisheries production in Korea

In 2013 approximately 3 million tons of seafood, valued of KRW 7.2 trillion (US$ 6.6 billion), were produced in Korea from capture fisheries (marine and inland) and aquaculture [16]. The volume of overall domestic fisheries production has stagnated since 2009 although within this there have been substantial changes with cultured seaweed production increasing significantly while fish production has greatly reduced (Table 1). Most change in volume of fish production has been from a few main species, notably squid, mackerel, and hairtail. This decline in catch appears to have come from several sources including overfishing, indirect ecosystem impacts, and illegal and unregulated catches from other nations within Korean waters [17]. In particular there was an estimated 0.7 million tons of catch from illegal fishing by Chinese registered vessels in the Korean Exclusive Economic Zone (EEZ) in 2012 [17]. This volume accounts for about 21% of total seafood production from the EEZ in the same year.

**Table 1:** Recent change in production (1000 t) of the upper 15 seafood taxa harvested from the Korean EEZ. *Source*: Statistics Korea (Each year).

2000		2007		2013	
Total yield	2514	Total yield	3275	Total yield	3135
Upper 15 taxa	1841	Upper 15 taxa	2441	Upper 15 taxa	2397
Squid	404	Squid	397	Red macroalgae	406
Sea mustard	212	Oysters	321	Kelp	373
Anchovy	201	Sea mustard	309	Sea mustard	327
Oyster	177	Kelp	250	Squid	255
Mackerel	145	Anchovies	221	Oyster	240
Bonito	137	Bonito	214	Anchovy	209
Red macroalgae	130	Red macroalgae	211	Bonito	201
Other fish	107	Mackerel	144	Mackerel	102
Pollock	86	Mussel	98	Hairtail	47
Hairtail	81	Hairtail	66	Herring	45
Yellowfin tuna	49	Yellowfin tuna	54	Yellowfin tuna	44
Croakers	31	Spanish mackerel	42	Krill	38
Bigeye tuna	30	Flatfish	41	Snow crab	38
Spanish mackerel	26	Krill	37	Flatfish	37
Pacific saury	25	Jacopever	36	Small yellow croaker	35

### Fisheries production in Korean offshore

The Korean General Marine Fishery has inshore and offshore components and contributed 51.9% of the total Korean domestic seafood production in 2013 [16]. From 2000, Ministry of Oceans and Fisheries in Korea has reduced the number of fishing vessels which have worked in General Marine Fishery for many reasons including EEZ declarations of adjacent countries, the fisheries agreements with adjacent countries, and responding to the depletion of stocks. As a result, the number of vessels has been reduced by 2.3% during the last three years so that 2,780 vessels operated in the Korean offshore fishery in 2013. Most vessels were in the size range of 20–50 tons (38.9%), followed by 50–100 tons (23.6%) and 10–20 tons (13.8%; Table 2). The main species produced by the offshore fishery were squid, hairtails, mackerel, and anchovies. A wide range of method categories were recorded with Danish trawl, pair trawl and otter trawl accounting for most yield, followed by gillnet, large purse seine, jigging and anchovy dragnet (Table 3).

**Table 2:** Vessel sizes in the Korean offshore fishery by method category in 2013. *Source*: Ministry of Oceans and Fisheries(MOF) Korea (2013).

Fishing Types	Total (M/T)	1–5	5–10	10–20	20–50	50–100	100–200	More than 200
Total (Vessels)	2,780	196	237	383	1,081	657	186	40
Large Danish Trawl	47	0	0	0	13	33	1	0
Large Pair Trawl	72	0	0	0	0	20	52	0
Eastern sea area Danish seine	38	0	0	0	10	28	0	0
Medium Size Danish Seine	39	0	0	0	26	13	0	0
Medium Size Pair Trawl	18	0	0	0	2	16	0	0
Large Trawler	52	0	0	0	0	0	52	0
Eastern Sea Area Otter Trawler	38	0	0	0	5	33	0	0
Large Purse Seine	143	0	0	0	0	47	56	40
Small Purse Seine	72	2	32	34	2	2	0	0
Jigging	480	0	21	70	288	94	7	0
Anchovy Dragnet	383	1	6	38	208	116	14	0
Gillnet	377	0	43	131	172	31	0	0
Stow Net	208	0	9	6	87	103	3	0
Lever Lift Net	3	0	3	0	0	0	0	0
Diver	235	188	47	0	0	0	0	0
Trap	200	0	10	19	58	112	1	0
Dredge	74	5	34	34	1	0	0	0
Long line	301	0	32	51	209	9	0	0

**Table 3:** Total production and main species in Korean offshore by method category in 2013. *Source*: Statistics Korea (2013).

Fishing Types	Yield (M/T)	Main taxa in catch
Large Danish Trawl	11,822	flatfish, brown croaker, blackthroat sea perch, blackmouth angler
Large Pair Trawl	55,758	hairtail, croaker, anchovy, silver pomfret, Spanish mackerel, gizzard shad
Eastern Sea Area Danish Seine	9,090	flatfish, sailfin sandfish
Medium Size Danish Seine	16,263	blackthroat seaperch, brown croaker, flatfish, blackmouth angler
Medium Size Pair Trawl	19,641	hairtail, anchovy, spanish mackerel, gizzard shad
Large Trawler	64,186	hairtail, squid
Eastern Sea Area Otter Trawler	37,052	squid
Large Purse Seine	163,856	hairtail, mackerel, yellowtail, Spanish mackerel, horse mackerel, sardine, herring, squid
Small Purse Seine	20,142	horse mackerel, mackerel, herring, anchovy
Jigging	42,567	hairtail, puffer, squid
Anchovy Dragnet	139,965	anchovy, spanish mackerel, herring
Gillnet	62,196	flatfish, pacific saury, cod, small yellow croaker, swimming crab, akiami paste shrimp, snow crab
Stow Net	46,611	hairtail, anchovy, silver pomfret, blackmouth angler, small yellow croaker, swimming crab, southern rough shrimp
Lever Lift Net	32	coral fish
Diver	8,678	Manila clam, Korean common penshell, egg cockle
Eel Fish Trap	8,874	white-spotted congor
Trap	39,555	swimming crab, snow crab, whelk, octopus
Dredge	160	Korean common penshell
Long Line	14855	hairtail, puffer

To investigate whether the offshore fisheries production varied by fishing type during last three years, a paired T-test was used to compare catch data from 2011–2012 and 2012–2013 (PASW Statistics Version 18.0). This did not reject the null hypothesis at the significance level of 0.1 levels, implying that there were no significant changes in production by method category during this three year period.

## Methods and Materials

### IPCC ‘Tier 1’ method

This study estimated GHG emissions from Korean offshore fisheries using Tier 1 of the three methods provided by the IPCC. Tier 1 is the basic method and it is designed to be used for all categories and is readily available nationally or internationally in combination with the IPCC default emission factors. The Tier 1 method can be applied with either default values or country-specific information. According to the IPCC, ‘Fishery’ is classified as water-borne navigation and mobile combustion, which requires the use of mobile rather than stationary emission factors [15]. The Water-Borne navigation calculation is based on the amount of fuel combusted and on emission factors for CO_2_, CH_4_, and N_2_O (Eq 1) [15]:

$$GHG\;Emissions\;=\;\sum \left(Fuel\;Consumed_{fuel\;type}\;\cdot \;Emission\;Factor_{fuel\;type}\right)$$ (1)

### Direct emissions of GHG

Water-borne navigation causes emissions of carbon dioxide (CO_2_), methane (CH_4_) and nitrous oxide (N_2_O), as well as smaller volumes of carbon monoxide (CO), non-methane volatile organic compounds (NMVOCs), sulphur dioxide (SO_2_), particulate matter (PM) and oxides of nitrogen (NO_x_) [15]. This study estimated the emissions of CO_2_, CH_4_ and N_2_O only as per the standard approach of the IPCC Tier 1 method. Estimating the emissions of these three compounds is considered to be sufficient to represent the full GHG emission because they are responsible for 95% of energy system emissions from the combustion of fossil fuels [15].

### Data source for estimate GHG Emissions

The Korean government has offered tax-free oil for all fishing vessels and the oil supply data is managed by National Federation of Fisheries Cooperatives (SUHYUP) [18] in Korea. This tax-free oil data was used to quantify total amount of fuel use in Korean offshore fisheries, as per previous research on fuel usage in this industry [19, 20, 21, 22]. The total number and size of vessels were obtained from the Fisheries Information Service in Korea [23], which is operated by the Ministry of Oceans and Fisheries. Catch data were sourced from Fisheries Production Statistics in Statistics Korea [16]. Emission factors using CO_2_, CH_4_ and N_2_O Emissions calculation were derived from IPCC (2006) [15] water-borne navigation factors as part of mobile combustion as Korea does not regional GHG emission factors for application. The amount of CH_4_ and N_2_O emissions were converted to Carbon Dioxide equivalents (CO_2_e) by multiplying each by their Global Warming Potential (GWP) and then combining with CO_2_ to derive the overall amount of GHG emission expressed as CO_2_e. GWP of each emission and Net Calorific Values by fuel type were based on the Greenhouse Gas Emissions Trading Act and the Energy Act standards in Korea [24]. This regional data was considered more accurate than global data supplied by the IPCC (Table 4).

**Table 4:** Global Warming Potential (GWP) by GHG and Net Calorific Values by fuel types used for offshore fishing vessels. Net Calorific Values used were revised values first published in December 2011. *Source*: Ministry of Government Legislation.

Emissions	Global Warming Potential (GWP)		
CO_2_	1		
CH_4_	21		
N_2_O	310		
Fuel Types	Unit	Net Calorific Values^a^	
		MJ (TJ/Gg)	Kcal
Gas/Diesel Oil	L	35.3	8,420
Residual Fuel Oil (B-A)	L	36.4	8,700
Residual Fuel Oil (B-B)	L	38.0	9,080
Lubricant	L	37.0	8,830

^a^ Revised December 2011

## Results and Discussion

### Status of fuel consumption by fishing types in offshore Korea

Fuel consumption of the fishery by method category was analysed using the last three years data (2011 to 2013) and tax-free oil sales data (Table 5). In 2013, the total tax-free oil consumption of the Korean fleet was 934,311 KL with 59.7% (557,463 KL) of this used by the offshore fleet. Thus, reduction in fuel use and GHG reductions in the offshore sector has a significant impact on the aggregate GHG emissions by Korean fisheries as it accounts for a significant proportion of the total.

**Table 5:** Annual tax-free oil consumption by method category in Korean offshore fisheries.

Fishing types	2011(KL)	2012	2013	Three-year average	Portion (%)
Total Domestic	919,349	900,380	934,311	918,013	
Total Offshore	544,185	533,371	557,463	545,006	100.0
Large Danish Trawl	14,968	15,121	15,542	15,210	2.8
Large Pair Trawl	58,994	54,283	61,223	58,167	10.7
Eastern Sea Area Danish	4,278	4,680	4,599	4,519	0.8
Medium size Danish Seine	12,445	12,072	13,079	12,532	2.3
Medium Size Pair Trawl	11,500	10,217	10,544	10,754	2.0
Large Trawler	38,427	32,008	34,922	35,119	6.4
Eastern Sea Area Otter Trawl	10,403	10,318	8,839	9,853	1.8
Large Purse Seine	85,685	86,119	87,553	86,452	15.9
Small Purse Seine	8,099	8,099	8,204	8,134	1.5
Jigging	76,494	79,083	84,757	80,111	14.7
Anchovy Dragnet	54,011	52,192	56,196	54,133	9.9
Gillnet	32,640	33,356	32,046	32,681	6.0
Stow net	40,257	40,795	41,893	40,982	7.5
Diver	4,242	4,023	3,661	3,976	0.7
Trap	47,793	49,778	52,941	50,171	9.2
Long Line	43,541	40,794	41,002	41,779	7.7
Others	406	423	464	431	0.1

The three-year average of the total oil consumption of the offshore fishing vessels in Korea was 545,006 KL p.a. with greatest usage by the Large Purse Seine Fishery, Jigging Fishery, Large Pair Trawl Fishery and Anchovy Dragnet Fishery. This usage was proportional to the tonnage of harvest and the size of vessels.

Trawl fisheries generally operated with large vessels so that annual fuel consumption per vessel was high (Table 6). Of these, the Large Pair Trawl Fishery had the highest annual fuel consumption per vessel, 827KL, based on 2011–2013 averages. This was followed by Large Trawler (680KL), Large Purse Seine Fishery (605 KL), and Medium Size Pair Trawl (587 KL).

**Table 6:** Tax-free oil consumption per vessel by method category in the offshore fishery based on 2011–2013 averages.

Fishing types	Three-year average			2013/2011(%)	
	Oil usage (KL)	Vessels	KL/Vessel	Vessels	KL/Vessel
Total Offshore Fisheries	545,006	2,822	193	97.7	102.4
Large Danish Trawl	15,210	47	324	100.0	103.8
Large Pair Trawl	58,167	70	827	102.9	103.8
Eastern Sea Area Danish	4,519	39	117	97.4	107.5
Medium size Danish Seine)	12,532	41	303	92.9	105.1
Medium Size Pair Trawl	10,754	18	587	94.7	91.7
Large Trawler	35,119	52	680	102.0	90.9
Eastern Sea Area Otter Trawl	9,853	39	255	97.4	85.0
Large Purse Seine	86,452	143	605	100.0	102.2
Small Purse Seine	8,134	73	111	97.3	101.3
Jigging	80,111	487	165	98.0	110.8
Anchovy Dragnet	54,133	387	140	97.5	104.0
Gillnet	32,681	379	86	99.7	98.2
Stow net	40,982	215	190	92.9	104.1
Diver	3,976	235	17	100.4	86.3
Trap	50,171	201	249	101.5	110.8
Long Line	41,779	315	132	93.2	94.2
Other	431	81	5	93.9	114.2

Importantly, aggregate fuel consumption increased despite a decrease in the number of vessels by 2.3% in 2013 compared to 2011 (Table 6). Two factors were identified that contributed to this: first the average annual fishing days increased slightly from 2011 to 2013 (186 to 190 days) [25]; and secondly, the average age of vessels operating in the fleet increased during this period, which is known to result in a decrease in combustion efficiency (Table 7) [26]. The number of older, less efficient vessels (over 16 years) has increased sharply while the number of younger vessels (under 10 years) reduced during this period (Table 7).

**Table 7:** Fishing vessels proportion by age in the Korean offshore fishery. *Source*: Ministry of Oceans and Fisheries(MOF) Korea.

Year	Total (Vessel)	Less than 5 years	6 to10 years	11 to 15 years	16 to 20 years	Over 21 years
2011	2,845	489	673	541	539	603
2012	2,842	471	634	545	567	625
2013	2,780	466	494	539	599	682

Analysis of fuel consumption by oil type over the last three years indicated that the oils used in offshore fisheries were diesel oil, residual fuel oil (B-A and B-B), blended oil (MF-30) and lubricant. Most fishing method categories used diesel oil (over 99%) while the Large Pair Trawl Fishery, Large Trawler, and Anchovy Dragnet Fishery used residual fuel oil at 15%, 41% and 14% respectively of total fuel usage. The consumption of MF-30 and lubricant in the vessels was less than 1% across all of the method categories [18].

### GHG emissions in offshore fisheries in Korea

Korean offshore fisheries GHG (CO_2_, CH_4_, N_2_O) emissions were calculated by applying IPCC Tier 1 method (Table 8). In the calculation, the net calorific value and the emission factors were applied differently by fuel types and the proportion of fuel used for the vessels of each fishing method category. Blended oil (MF-30) and lubricant were replaced by diesel oil’s emission factor and net calorific value as their proportion was less than 1%.

**Table 8:** GHG emissions by fishing method category in the Korean offshore fishery.

Fishing types	In 2011(tCo_2_e)	In 2012	In 2013	Three-year average
Total Offshore Fisheries	1,442,975	1,412,353	1,477,279	1,444,202
Large Danish Trawl	39,479	39,883	40,992	40,118
Large Pair Trawl	157,560	144,977	163,514	155,350
Eastern Sea Area Danish Seine	11,284	12,343	12,130	11,919
Medium size Danish Seine	32,826	31,841	34,496	33,055
Medium Size Pair Trawl	30,333	26,948	27,810	28,363
Large Trawler	104,841	87,326	95,278	95,815
Eastern Sea Area Otter Trawl	27,439	27,215	23,313	25,989
Large Purse Seine	226,002	227,146	230,929	228,026
Small Purse Seine	21,361	21,362	21,638	21,454
Jigging	201,760	208,589	223,555	211,301
Anchovy Dragnet	144,652	138,523	149,937	144,370
Gillnet	86,091	87,980	84,524	86,198
Stow net	106,182	107,601	110,497	108,093
Diver	11,190	10,612	9,657	10,486
Trap	126,059	131,294	139,637	132,330
Long Line	114,845	107,597	108,147	110,196
Others	1,072	1,116	1,225	1,138

It was apparent that GHG emissions generated by the aggregate offshore fisheries increased from 1,442,975 tCO_2_e/year in 2011 to 1,477,279 tCO_2_e/year in 2013. In 2012, the GHG emissions were lower than in 2011 because in 2012 the average annual fishing days in offshore fisheries decreased by approximately 6% compared to 2011. This was due to a temporary spike in international oil prices from a EU ban on oil imports from Iran as a sanction for Iran's nuclear development in 2012 [27]. The amount of GHG emissions for the last three-year average was identified at 1,444,202 tCO_2_e/year.

Comparison of GHG emissions across fishing method categories showed that the Large Purse Seine Fishery generated the highest GHG emissions followed by the Jigging Fishery and the Large Pair Trawl Fishery. At the other extreme, GHG emissions from the Diver Fishery, Eastern Sea Area Danish Seine and Small Purse Seine Fishery were low relative to other fishing methods.

GHG emissions per vessel and per production of 1kg fish varied by fishing type and were highest in in 2013 due to change in the number of fishing days and also change in efficiency of vessels (Table 9). The GHG emissions per kg of fish increased for each of the three consecutive years due to the decrease in catch rate during this period so that by 2013, GHG emissions per kg fish product were 16% higher than in 2011. Over this three-year period, catch rate had an overall decline of 10.6% from 2011 (306.3 t/vessel) to 2013 (273.9 t/vessel) [28, 29]. Although this change in catch rate and resultant increase in GHG emissions was clearly not desirable, it does illustrate that if decline in catch rate was reversed there would be substantial benefits for fuel consumption and reduction of GHG emissions.

**Table 9:** GHG emissions per vessel and by production (per kg fish) by fishing method category in the Korean offshore fishery.

Fishing types	GHG emissions per vessel (tCo_2_e/ vessel)			GHG emissions per production (kg Co_2_e/ kg fish)		
	In 2011	In 2012	In 2013	In 2011	In 2012	In 2013
Total Offshore fisheries	507	497	531	1.67	1.79	1.94
Large Danish Trawl	840	849	872	3.20	3.38	3.47
Large Pair Trawl	2,251	2,101	2,271	2.63	2.68	2.93
Eastern Sea Area Danish	289	316	319	1.84	1.82	1.33
Medium size Danish Seine	782	740	885	2.13	2.06	2.12
Medium Size Pair Trawl	1,596	1,497	1,545	1.27	1.03	1.42
Large Trawler	2,056	1,679	1,832	1.51	1.13	1.48
Eastern Sea Area Otter Trawl	704	698	613	.56	.66	.63
Large Purse Seine	1,580	1,588	1,615	1.03	1.28	1.41
Small Purse Seine	289	289	301	1.06	1.05	1.07
Jigging	412	426	466	4.48	4.22	5.25
Anchovy Dragnet	368	359	391	.94	1.04	1.07
Gillnet Fishery	228	231	224	1.18	1.45	1.36
Stow net	474	503	531	2.12	2.43	2.37
Diver	48	45	41	1.14	1.18	1.11
Trap	640	634	698	3.01	2.75	2.88
Long Line	356	334	359	6.88	7.02	7.28

Higher catch rates can be achieved through effective management of catch and this produces far greater proportional changes in emissions than modifications to engines or gear [30]. We note that implicit in this observation is the assumption that higher abundance of stock will translate into higher catch rates and thus reduced fuel consumption. Other factors modifying the relationship between stock abundance and fuel consumption are temporal changes in catchability of the stock and changes to gear selectivity. Many of the target species in the Korean offshore fishery are schooling and this behavior tends to mask the effect of changes in abundance on catch rate.

The Large Pair Trawl Fishery generated the highest per vessel GHG emission of all fisheries in 2013 followed by vessels in the Large Trawler and Large Purse Seine Fishery method categories. This was nearly identical to trends in tax-free oil consumption by fishing method category (Table 5), however there was divergence in the trend between tax-free oil consumption and GHG emissions per kg of production for the Long Line Fishery, Jigging Fishery and Large Danish Trawl Fishery with disproportionately higher emissions in each case.

## Conclusion

This quantitative study assessed the fuel use and the GHG emissions from the offshore fisheries, which dominate domestic seafood production in Korea.

Fuel usage by offshore fisheries in 2013 accounted for 59.7%(557,463 KL) of total fuel consumption of fishing vessels in Korea which shows that changes in these fisheries will have a significant impact on fuel use and GHG emissions for the entirety of the Korean fisheries. Those fisheries that used larger vessels (Large Pair Trawl Fishery, Large Trawler and Large Purse Seine Fishery) had higher fuel consumption and higher fuel per vessel among all offshore fishing types over the last three-years, from 2011 to 2013 on average. The larger scale of vessels operating with these method categories did not appear to result in substantial gains in efficiency in terms of emissions per kg of catch with many other method categories having lower emissions per kg of catch, including small purse seine and medium sized pair trawl.

Another important result from this study was the 2.4% increase in fuel consumption that has occurred despite a decrease in the number of vessels in each of the consecutive years. Although this is small in scale, we are confident that this increase represents a real change because fuel consumption data is collected at the point of supply and monitored closely by the SUHYUP because it has implications for tax-free status of this fuel.

The increase in fuel consumption showed that the fuel efficiency of vessels in the offshore fleet deteriorated each year, which increases GHG emissions independently of change in catch rate. Decline in efficiency of vessels was most apparent from those fisheries that had larger sized vessels where aging of the vessels appeared to have affected fuel combustion efficiency. In 2013, the highest proportion of older vessels (over 16 years) was in the Large Purse Seine Fishery 98.6%, Large Trawler 92.3%, Eastern Sea Area Danish 89.5% and Large Danish Trawl Fishery 89.4% method categories. These four categories contributed around one third of the offshore fishery production so this decline in fuel consumption efficiency from 2011 to 2013 would significantly impact the GHG emissions of the overall Korean fishery if efficiency continued to deteriorate. Reduced efficiency should create an economic incentive for fishers to renew engines but if this fails there may be a need for policy or additional incentives to promote engine replacement.

Our results also showed that GHG emissions generated by the entire offshore fisheries increased from 1,442,975 tCO_2_e/year in 2011 to 1,477,279 tCO_2_e/year in 2013. In 2013, the Large Purse Seine Fishery generated the highest GHG emissions of all the offshore fisheries, closely followed by Jigging Fishery. A factor contributing to the increase in emissions across the fleet was that the GHG emissions per kg of production increased every year due to decline in catch rate. This decline in catch rate is consistent with a decrease in fish abundance, with concerns about the status of fish stocks expressed previously by the National Fisheries Research and Development Institute Korea [31] due to deterioration of coastal habitats and excessive catch, resulting in an overall decline of 10.6% [28, 29]. Decrease in fish abundance results in an increase in fishing effort to obtain the same output with associated increase in the fishing vessels’ working time, fuel consumption and GHG emissions [32].

Although decline in fish stocks appear to have contributed to an increase in fuel consumption in the Korean offshore fisheries, there is also a positive aspect to this result because it shows that management of fish harvests and thus fish stocks provides a method for controlling GHG emissions. Rebuilding of stocks through tighter controls on catch also provides benefits to fishing industries of greater economic yield and more stable production across years [33, 34]. This interaction between management decisions directed to fishery catch controls and GHG emissions was explored by Farmery et al. [28] who showed that targeting higher stock abundance and sustainable higher economic yield from fisheries can reduce the GHG emissions substantially. They estimated a reduction in GHG emissions of 80% through shifting the management target from MSY(Maximum Sustainable Yield) to MEY(Maximum Economic Yield).

This analysis also showed that GHG emissions per kg offshore fish production was highly variable between fishing method category and was very high for some. The Long Line Fishery was more than three times the average and Jigging Fishery more than two times higher than the average for the total offshore fisheries. This suggests that the Korean government has opportunity to reduce overall emissions by managing the catch allocation between different fishing method categories. This is clearly complicated however as management and policy needs to balance between competing objectives. In these two particular fisheries, jigging supplies catch from squid that are difficult to harvest by other methods, while long lining is often seen as a more ecologically preferable to dragged gears such as trawl despite the higher emissions shown here for long lining.

This study was restricted to an analysis of the GHG emissions of offshore fishery. Expansion to the total Korean fisheries including deep-sea and coastal fisheries in the future would allow a more integrated analysis of GHG emissions for Korean fisheries. Our analysis detected important changes over the three-year period where data were available so future analysis with a longer term dataset would be helpful to determine if changes in efficiency and GHG emissions were continuing in Korean fisheries.
